# Gender differences in the temporal voice areas

**DOI:** 10.3389/fnins.2014.00228

**Published:** 2014-07-30

**Authors:** Merle-Marie Ahrens, Bashar Awwad Shiekh Hasan, Bruno L. Giordano, Pascal Belin

**Affiliations:** ^1^Centre for Cognitive Neuroimaging, Institute of Neuroscience and Psychology, University of GlasgowGlasgow, UK; ^2^Institut des Neurosciences de la Timone, UMR 7289, CNRS and Université Aix-MarseilleMarseille, France; ^3^International Laboratories for Brain, Music and Sound, Department of Psychology, Université de Montréal, McGill UniversityMontreal, QC, Canada

**Keywords:** gender difference, fMRI, voice localizer, temporal voice areas, multivariate pattern analysis (MVPA), voice perception

## Abstract

There is not only evidence for behavioral differences in voice perception between female and male listeners, but also recent suggestions for differences in neural correlates between genders. The fMRI functional voice localizer (comprising a univariate analysis contrasting stimulation with vocal vs. non-vocal sounds) is known to give robust estimates of the temporal voice areas (TVAs). However, there is growing interest in employing multivariate analysis approaches to fMRI data (e.g., multivariate pattern analysis; MVPA). The aim of the current study was to localize voice-related areas in both female and male listeners and to investigate whether brain maps may differ depending on the gender of the listener. After a univariate analysis, a random effects analysis was performed on female (*n* = 149) and male (*n* = 123) listeners and contrasts between them were computed. In addition, MVPA with a whole-brain searchlight approach was implemented and classification maps were entered into a second-level permutation based random effects models using statistical non-parametric mapping (SnPM; Nichols and Holmes, 2002). Gender differences were found only in the MVPA. Identified regions were located in the middle part of the middle temporal gyrus (bilateral) and the middle superior temporal gyrus (right hemisphere). Our results suggest differences in classifier performance between genders in response to the voice localizer with higher classification accuracy from local BOLD signal patterns in several temporal-lobe regions in female listeners.

## Introduction

Prior functional magnetic resonance imaging (fMRI) findings suggest a robust brain response to vocal vs. non-vocal sounds in many regions of the human auditory cortex in particular in the superior temporal gyrus (STG). Vocal sounds, including but not restricted to speech sounds, evoke a greater response than non-vocal sounds with bilateral activation foci located near the anterior part of the STG extending to anterior parts of the superior temporal sulcus (STS) and posterior foci located in the middle STS (Binder et al., 2000; Belin et al., 2000, 2002). Using the functional voice localizer, these findings were replicated and used in various studies (Belin et al., 2000, 2002; Kreifelts et al., 2009; Latinus et al., 2011; Ethofer et al., 2012). The conventional way of identifying voice sensitive regions is by applying univariate statistics, implemented using a Generalized-Linear Model (GLM), to fMRI data assuming independence among voxels.

Interest has recently grown in applying multivariate approaches (e.g., Multivariate pattern analysis; MVPA). Instead of modeling individual voxels independently (univariate analysis), MVPA considers the information of distributed pattern in several voxels (e.g., Norman et al., 2006; Mur et al., 2009). Several studies used multivariate approaches to decode information reflected in brain activity patterns related to specific experimental conditions (Cox and Savoy, 2003; Haynes and Rees, 2005, 2006; Kotz et al., 2013). MVPA is usually applied on unsmoothed data preserving high spatial frequency information. Thus, MVPA is argued to be more sensitive in detecting different cognitive states. In contrast, the conventional univariate analysis averages across voxels, thereby removing focally distributed effects (spatial smoothing). The smoothing across voxels may lead to a reduction in the information content (Kriegeskorte et al., 2006; Norman et al., 2006; Haynes et al., 2007). At present, a multivariate approach has never been employed to investigate whether it may yield a different pattern of voice-specific (voice/non-voice classification) brain regions compared to the univariate analysis.

The voice contains socially and biologically relevant information and plays a crucial role in human interaction. This information is particularly relevant for interaction between different genders (e.g., regarding emotions, identities, and attractiveness) (Belin et al., 2004, 2011). Overall, research suggests that women are more sensitive than men in emotion recognition from faces and voices (Hall, 1978; Hall et al., 2006; Schirmer and Kotz, 2006). Women perform better in judging others' non-verbal behavior (Hall, 1978) and seem to process nonverbal emotional information more automatically as compared to men (Schirmer et al., 2005). In addition, women but not men show greater limbic activity when processing emotional facial expressions (Hall et al., 2004). The exact neural mechanisms underlying voice processing in both female and male listeners still remains under debate. For instance, a study by Lattner et al. (2005) found no significant difference between the activation patterns of female and male listeners in response to voice-related information. However, there is evidence from both behavioral and neural activation studies for differences in voice perception between listeners' gender (Shaywitz et al., 1995; Schirmer et al., 2002, 2004, 2007; Junger et al., 2013; Skuk and Schweinberger, 2013).

A recent behavioral study by Skuk and Schweinberger (2013) investigated gender differences in a familiar voice identification task. They found an own-gender bias for males but not for females while females outperformed males overall. These behavioral differences (Skuk and Schweinberger, 2013) may also be reflected by differences in neural activity. Previous fMRI studies investigating potential neural correlates suggested a sex difference in the functional organization of the brain for phonological processing (Shaywitz et al., 1995), in emotional prosodic and semantic processing (Schirmer et al., 2002, 2004) and in response to gender-specific voice perception (Junger et al., 2013). Further evidence suggests differences between genders in vocal processing shown by an EEG study, where the processing of vocal sounds with more emotional and/or social information was more sensitive in women as compared to men (Schirmer and Kotz, 2006; Schirmer et al., 2007). The above-mentioned studies mainly focus on gender differences in emotional speech processing or opposite-sex perception. However, identified brain regions are not consistent: different experimental designs and applied methods vary and make it difficult to compare between these studies (Shaywitz et al., 1995; Schirmer et al., 2002, 2004, 2007; Junger et al., 2013).

The current study employs a well-established experimental design of the functional “voice localizer,” known to give robust estimates of the TVAs across the majority of participants. The voice localizer includes a variety of different vocal sounds, not exclusively female or male voices, but also speech and non-speech of women, men and infants and non-vocal sounds (e.g., environmental sounds). In this study, we were interested in the effect of gender on the results of the voice localizer and we asked an explorative research question of whether brain activation and/or classification accuracy maps in response to vocal (speech and non-speech) and non-vocal sounds differ between female and male listeners without prior assumptions about the strength of voice-specific activity.

The voice localizer paradigm is often used in the literature (Belin et al., 2000, 2002; Kreifelts et al., 2009; Latinus et al., 2011; Ethofer et al., 2012), which makes it easier to compare among studies as well as among participants or groups. Instead of using the conventional univariate method, employing MVPA may offer a more sensitive approach in order to study potential differences between genders by means of above chance vocal/non-vocal classification accuracies in different regions of the brain. Therefore, we investigated our research question by implementing the conventional univariate analysis using GLM and MVPA based on a support-vector machine (SVM) classifier with a spherical searchlight approach. This approach enabled us to explore cortical activity over the whole-brain and to examine whether activation and/or classification maps in response to the voice localizer may significantly differ between genders. Since the effect size between genders is expected to be very small, the current study offers a substantially large sample size with *n* = 149 females and *n* = 123 males. Thus, this study provides a large sample size, a well-established experimental design and the direct comparison of two different fMRI data analysis approaches applied on the exact same data.

## Methods

### Participants

fMRI data of 272 healthy participants, 149 female (age range: 18–68 years; mean ± *SD* = 24.5 ± 8.0) and 123 male (age range: 18–61 years; mean ± *SD* = 24.4 ± 6.5) with self-reported normal audition were analyzed. This study was conducted at the Institute of Neuroscience and Psychology (INP) in Glasgow and approved by the ethics committee of the University of Glasgow. Volunteers provided written informed consent before participating and were paid afterwards.

### Voice localizer paradigm

Subjects were instructed to close their eyes and passively listen to a large variety of sounds. Stimuli were presented in a simple block design and divided into vocal (20 blocks) and non-vocal (20 blocks) conditions. Vocal blocks contained only sounds of human vocal origin (excluding sounds without vocal fold vibration such as whistling or whispering) and consisted of speech (e.g., words, syllables, connected speech in different languages) or non-speech (e.g., coughs, laughs, sighs and cries). The vocal stimuli consisted of recordings from 7 babies, 12 adults, 23 children, and 5 elderly people. Half of the vocal sounds (speech and non-speech) consisted of vocalizations from adults and elderly people (women and men) with comparable proportions for both genders (~24% female, ~22% male). The other half of the vocal sounds consisted of infant vocalizations (speech and non-speech) which also included baby crying/laughing. Recorded non-vocal sounds included various environmental sounds (e.g., animal vocalizations, musical instruments, nature and industrial sounds). A total number of 40 blocks were presented. Each block lasted for 8 s with an inter-block interval of 2 s. Stimuli (16bit, mono, 22050 Hz sampling rate) were normalized for RMS and are available at http://vnl.psy.gla.ac.uk/resources.php (Belin et al., 2000).

### MRI data acquisition

Scanning was carried out in a 3T MR scanner (Magnetom Trio Siemens, Erlangen, Germany) and all data were acquired with the same scanner at the INP in Glasgow. Functional MRI volumes of the whole cortex were acquired using an echo-planar gradient pulse sequence (voxel size = 3 mm × 3 mm × 3 mm; Time of Repetition (TR) = 2000 ms; Echo Time (TE) = 30 ms; slice thickness = 3 mm; inter-slice gap = 0.3 mm; field of view (FoV) = 210 mm; matrix size = 70 × 70; excitation angle = 77°). A total number of 310 volumes (32 slices per volume, interleaved acquisition order) were collected with a total acquisition time of 10.28 min. Anatomical MRI volumes were acquired using a magnetization-prepared rapid gradient echo sequence (MPRAGE) (voxel size = 1 × 1 × 1 mm; *TR* = 1900 ms; *TE* = 2.52 ms; inversion time (TI) = 900 ms; slice thickness = 1 mm; FoV = 256 mm; matrix size = 256 × 265; excitation angle = 9°; 192 axial slices).

### fMRI data analysis

#### Pre-processing

Pre-processing was performed using the statistical parametric mapping software SPM8 (Department of Cognitive Neurology, London, UK. http://www.fil.ion.ucl.ac.uk/spm/software/spm8/). After reorientation of functional and anatomical volumes to the AC/PC line (anterior- and posterior commissure), functional images were motion corrected (standard realignment). Since, subjects may have moved between anatomical and functional data acquisition, the anatomical volumes were co-registered to the mean functional image produced in the realignment above. Anatomical volumes were segmented in order to generate a binary gray matter template at threshold probability level of 0.5 for each individual participant. This template was applied during model specification in both univariate analysis und MVPA. For the univariate processing, realigned functional volumes were normalized to a standard MNI template (Montreal Neurological Institute) and spatially smoothed with a 6 mm full-width at half mean (FWHM) Gaussian Kernel.

#### Univariate analysis

The design matrix was defined such that each block of the experimental paradigm correlated to one condition, yielding a design matrix with 20 onsets for each condition (vocal and non-vocal). Analysis was based on the conventional general linear model (GLM) and stimuli were convolved with a boxcar hemodynamic response function provided by SPM8. Contrast images of vocal vs. non-vocal conditions were generated for each individual subject and entered into a second-level random effects analysis (RFX). To declare at the group-level whether any difference between the two conditions was significantly larger than zero, a one-sample *t*-test was applied and FWE-corrected (*p* < 0.05) brain maps were calculated. To investigate whether brain activity significantly differs between genders in response to vocal vs. non-vocal sounds, contrasts between females vs. males (male > female, female > male) were computed in a second level RFX analysis (two-sample *t*-test; *p* < 0.05 FWE-corrected). This analysis was restricted to voxels with classification accuracy significantly above theoretical chance (*p* < 0.01 uncorrected) in both females and males (see MVPA below and yellow area in **Figure 2**).

#### Multivariate pattern analysis

Multivariate pattern classification was performed on unsmoothed and non-normalized data using Matlab (Mathworks Inc., Natick, USA) and in-house utility scripts (INP, Voice Neurocognition Laboratory; Dr. Bashar Awwad Shiekh Hasan and Dr. Bruno L. Giordano), where the default linear support vector machine (SVM) classifier was applied. The classifier was trained and separately tested following a leave-one out cross validation strategy applied on the 40 beta parameter estimates obtained from the univariate analysis (GLM).

A whole-brain searchlight decoding analysis was implemented using a sphere with a radius of 6 mm (average number of voxels in one sphere: 20.6 ± 1.0 *SD*) (Kriegeskorte et al., 2006). A sphere was only considered for analysis if a minimum of 50% of its voxels were within the gray matter. The data of the voxels within a sphere were classified and the classification accuracy was stored at the central voxel, yielding a 3D brain map of classification accuracy (percentage of correct classifications) (Kriegeskorte et al., 2006; Haynes et al., 2007). To identify brain regions in which classification accuracy was significantly above chance by females and males, the theoretical chance level (50%) was subtracted, then normalized (to the MNI template) and smoothed (6 mm FWHM Gaussian Kernel). To make inference on female and male participants, classification brain maps were entered into a second-level permutation based analysis using statistical nonparametric mapping (SnPM; Statistical NonParametric Mapping; available at http://warwick.ac.uk/snpm) with 10,000 permutations (see Holmes et al., 1996; Nichols and Holmes, 2002). This was computed separately by gender and the resulting voxels were assessed for significance at 5% level and FWE-corrected, as determined by permutation distribution. Similarly, to assess whether classification brain maps significantly differ between genders in response to vocal/non-vocal sounds, this permutation approach was implemented between groups (female > male, male > female) with 10,000 permutations and the resulting voxels were assessed for significance at 5% level and FWE-corrected, as determined by permutation distribution (see Holmes et al., 1996; Nichols and Holmes, 2002).

The between-group analysis was restricted to a mask defined by voxels with classification accuracy significantly above theoretical chance (*p* < 0.01 uncorrected) in both females and males. The resulting mask included 3783 voxels (yellow area in **Figure 2**). The same mask was applied for both, the univariate analysis and MVPA.

Separate brain maps of vocal vs. non-vocal contrast in female and male participants as well as brain maps of contrasts between genders for both, univariate analysis and MVPA were generated using the program MRIcoGL (available at http://www.mccauslandcenter.sc.edu/mricro/mricron/).

## Results

### Univariate analysis: vocal vs. non-vocal sounds

**Figure 1:**
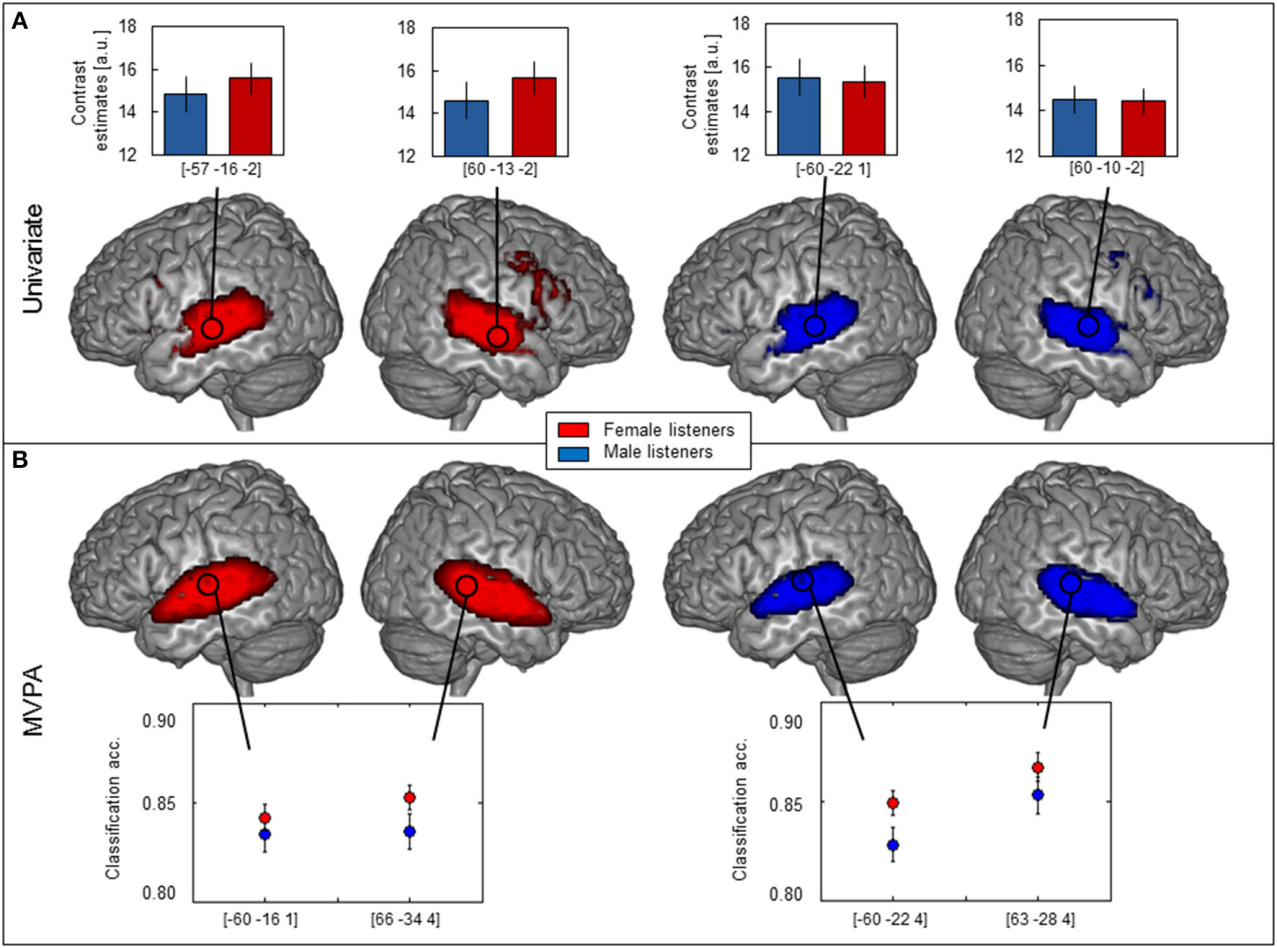
**Brain maps of female (red, *n* = 149) and male (blue, *n* = 123) participants**. **(A)** Univariate analysis showing bilateral activation along the superior temporal sulcus (STS) and in the inferior frontal gyrus (IFG) and corresponding contrast estimates of vocal vs. non-vocal sounds plotted for peak voxel (one-sample *t*-test, FWE-corrected, *p* < 0.05; cf. circles, note that the two peaks with highest *T*-value and largest cluster size are indicated per group). **(B)** MVPA showing comparable classification accuracy maps along STS, but not IFG and average classification accuracy ± s.e.m. at peak voxel (calculated in native space) was distinctly above chance level (0.5) for both females and males (maximum intensity projection of *t*-statistic image threshold at FWE-corrected *p* < 0.05, as determined by permutation distribution with 10,000 permutations).

The univariate analysis comparing activation to vocal and non-vocal sounds showed extended areas of greater response to vocal sounds in the typical regions of the temporal voice areas (TVA), highly similar for male and female subjects (Figure 1A). These regions were located bilaterally in the temporal lobes extending from posterior parts of the STS along the STG to anterior parts of the STS and also including several parts of the superior and middle temporal gyrus (STG, MTG).

Several hemispheric maxima of vocal vs. non-vocal response were located bilaterally along the STS in both females and males (Figure 1, Table 1). Figure 1A shows parameter estimates of the vocal > non-vocal contrasts at the maxima of the largest cluster sizes with the highest *T*-values of each hemisphere. The brain activation differences between vocal and non-vocal response was consistent across maxima in females (MNI coordinates left: *x* = −57, *y* = −16, *z* = −2, cluster size 3923, *T* = 20.85; right: *x* = 60, *y* = −13, *z* = −2, *T*-value = 20.64) and in males (MNI coordinates left: *x* = −60, *y* = −22, *z* = 1, cluster size 796, *T* = 18.19; right: *x* = 60, *y* = −10, *z* = −2, cluster size 812, *T*-value = 17.46). Female listeners showed one large cluster covering the temporal lobes and subcortical parts of the brain. By contrast male listeners showed two separate voxel clusters in the left and right temporal lobes and no subcortical cluster connecting the two hemispheres (Table 1). Small bilateral clusters were found in inferior prefrontal cortex (inferior frontal gyrus, IFG) in both female and male listeners (*p* < 0.05 FWE-corrected; Figure 1A).

**Table 1:** **Voice-sensitive peak voxels of female and male RFX analysis (Univariate)**.

**Anatomical location**	**Peak voxel *x*, *y*, *z***	***t*-values**	**Cluster size**
**FEMALE LISTENERS**			
**Left/Right hemisphere**			
**Left STG, middle**	−57, −16, −2	20.85	3923
Right STG, middle	60, −13, −2	20.64	
Right STG, middle	63, −22, −2	20.11	
**Left frontal hemisphere**			
**IFG (pars triangularis)**	−48, 17, 22	9.25	178
IFG (pars triangularis)	−39, 29, −2	8.79	103
Precentral gyrus	−48, −7, 43	6.32	5
**Right frontal hemisphere**			
**IFG (orbital)**	48, 17, −8	4.87	1
**MALE LISTENERS**			
**Left hemisphere**			
**STG, middle**	−60, −22, 1	18.15	796
STG, middle	−57, −13, −2	17.97	
STG, posterior	−60, −37, 4	12.73	
**IFG (pars triangularis)**	−42, 29, −2	7.96	40
**IFG (pars triangularis)**	−42, 17, 22	5.56	32
**Hippocampus**	−18, −10, −14	4.61	1
**Right hemisphere**			
**STG, middle**	60, −10, −2	17.40	812
STG, middle	63, −22, −2	17.11	
STG, anterior	54, 5, −14	11.51	
**IFG (pars triangularis)**	42, 32, −2	6.76	165
IFG (pars triangularis)	54, 23, 22	6.62	
IFG (pars triangularis)	45, 17, 22	6.51	
**Precentral gyrus**	51, −1, 46	7.60	22

Peak voxel coordinates in standard MNI space and corresponding t-values above for female and male 4.49 (FWE-corrected p < 0.05).

### MVPA analysis: vocal/non-vocal classification

The MVPA analysis showed clusters of significantly above-chance voice/non-voice classification accuracy in the TVAs (Figure 1B, Table 1) (Figure 1A, Table 2). Hemispheric maxima of classification accuracy were at comparable locations as the peaks of voice > non-voice activation revealed by the univariate method. The classification accuracy within the peak voxel of female listeners (MNI coordinates left: *x* = −60, *y* = −16, *z* = 1, cluster size 1676, *T*-value = 20.41; right: *x* = 66, *y* = −31, *z* = 4, cluster size 1671, *T*-value = 21.45) as well as for male listeners (MNI coordinates left: *x* = −60, *y* = −22, *z* = 4, cluster size 984, *T*-value = 13.70; right: *x* = 63, *y* = −28, *z* = 4, cluster size 1211, *T*-value = 16.07) were distincly above the theoretical chance level of 0.5 (Figure 1B). Overall, the maximal classification accuracy was higher in female listeners as compared to male listeners at the peak voxels (Figure 1B, mean ± s.e.m.: left peak in females 0.84 ± 0.006, males 0.83 ± 0.009; right peak in females 0.85 ± 0.007, males 0.84 ± 0.009. Left peak in males 0.83 ± 0.009, females 0.85 ± 0.006, right peak in males 0.85 ± 0.009, females 0.87 ± 0.007). Comparing MVPA and univariate analysis in Figures 1A,B the MVPA analysis revealed more superficial cortical regions bilateral at the temporal pole, whereas the voxel cluster of the vocal vs. non-vocal difference of the univariate analysis extend more toward the midline of the brain.

**Table 2:** **Voice-sensitive peak voxels of female and male group analysis (MVPA)**.

**Anatomical location**	**Peak voxel *x*, *y*, *z***	***t*-values**	**Cluster size**
**FEMALE LISTENERS**			
**Left hemisphere**			
**MTG, anterior**	−60, −16, 1	20.41	1676
MTG, posterior	−63, −37, 7	18.26	
**Right hemisphere**			
**STG, middle**	66, −31, 4	21.45	1671
STG, anterior	60, −7, −5	19.49	
**MALE LISTENERS**			
**Left hemisphere**			
**MTG, middle**	−60, −22, 4	13.70	984
**Right hemisphere**			
**STG, middle**	63, −28, 4	16.07	1211
MTG, anterior	63, −10, −5	14.88	

Peak voxel coordinates in standard MNI space and corresponding t-values above for female 4.38 and male 4.29 (FWE-corrected p < 0.05, as determined by permutation distribution with 10,000 permutations).

### Female vs. male contrasts

**Figure 2:**
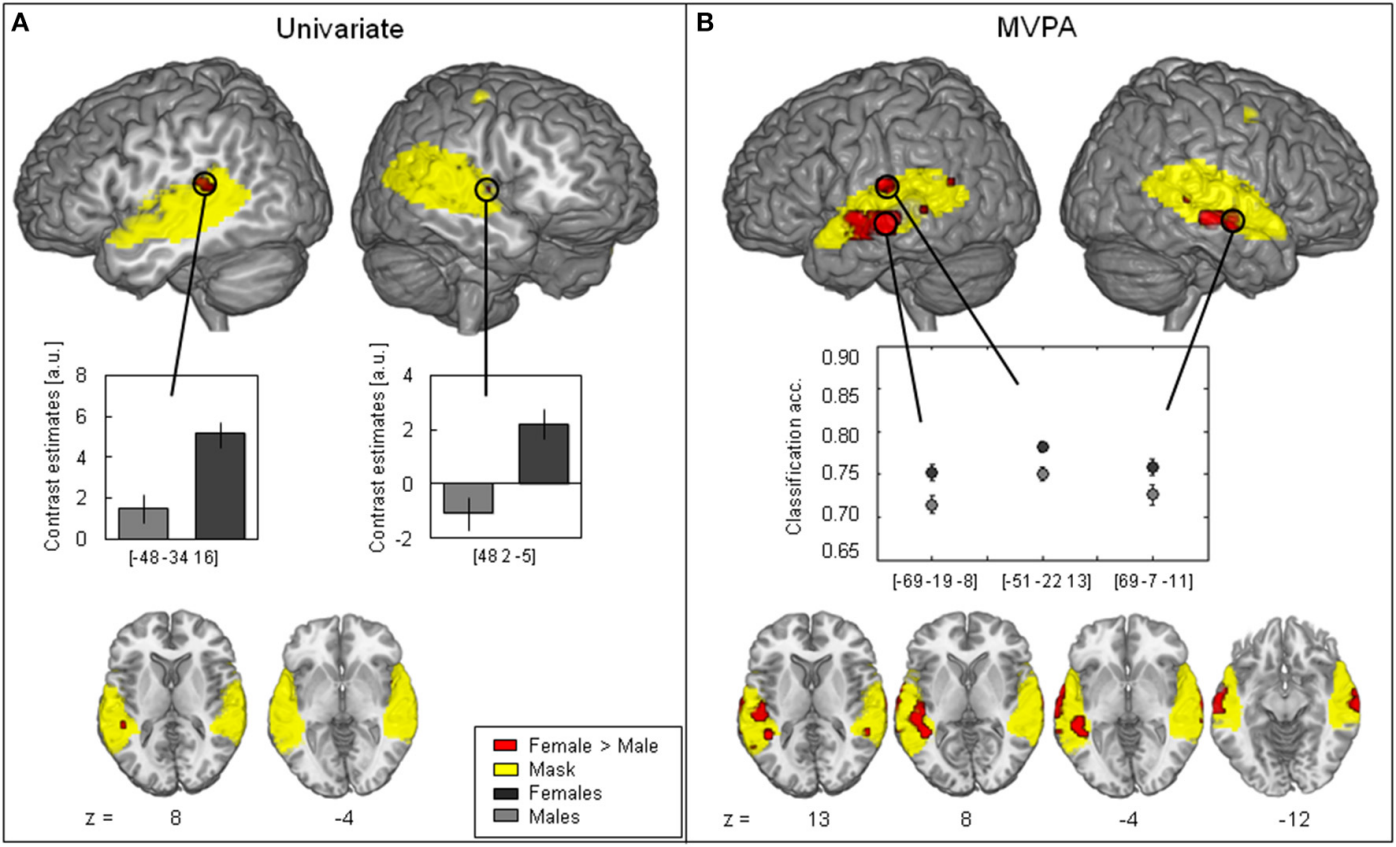
**Contrast between female > male (red)**. **(A)** Univariate analysis showing significant female > male difference (two-sample *t*-test, FWE-corrected, *p* < 0.05) in the left posterior part of the superior temporal gyrus (STG) and the right anterior STG. Contrast estimates at peak voxel showing stronger activation in females (black) as compared to males (gray) in response to vocal vs. non-vocal sounds. **(B)** MVPA showing significant classification accuracy above chance level in the right middle part of the middle temporal gyrus (MTG) and the right middle STG as well as in the left middle MTG with higher average classification accuracy in females (black) than in males (gray) (maximum intensity projection of *t*-statistic image threshold at FWE-corrected *p* < 0.05, as determined by permutation distribution with 10,000 permutations). The (yellow) cluster shows the mask including voxels with significantly above chance classification accuracy in both females and males (*p* < 0.01 uncorrected).

The contrast of activation maps (univariate analysis) or classification accuracy maps (multivariate approach) from males and females revealed no significant voxels with greater parameter estimates for males > females at the chosen statistical significance threshold (*p* < 0.05, FWE-corrected) for either analysis methods. The reverse contrast (female > male), however, revealed significant voxel clusters showing greater parameter estimates for univariate analysis and higher classification accuracy for MVPA in female participants (Figure 2).

When analyzed with the univariate approach (Figure 2A) the contrast female > male yielded only a few significant voxels: One cluster consisted of four voxels in the left posterior part of STG and only one voxels in the right Insula (Figure 2A, Table 3). The corresponding contrast estimates for the reported peak voxels (MNI coordinates left: *x* = −48, *y* = −34, *z* = 16, cluster size 4, *T*-value = 4.02; right: *x* = 48, *y* = 2, *z* = −5, cluster size = 1, *T*-value = 4.04) showed a positive response for females in both hemispheres and for the left hemisphere in males. The Cohen's d effect size values (*d* = 0.48 and 0.49) suggested a moderate difference at the peak voxel (Table 3). Overall, females showed a stronger activation in response to vocal vs. non-vocal sounds as compared to males at both maxima (Figure 2A).

**Table 3:** **Peak voxels of female > male contrast for univariate analysis and MVPA**.

**Anatomical location**	**Peak voxel *x*, *y*, *z***	***t*-values**	**Cluster size**	**Cohen's d at the peak voxel**
**UNIVARIATE (FEMALE > MALE)**				
**Left hemisphere**				
**STG, posterior**	−48, −34, 16	4.02	4	0.48
**Right hemisphere**				
**Insula**	48, 2, −5	4.04	1	0.49
**MVPA (FEMALE > MALE)**				
**Left hemisphere**				
**STG, middle**	−69, −19, −8	5.22	84	0.35
STG, middle	−66, −1, −8	5.02		
**STG, middle**	−51, −22, 13	5.19	156	0.35
STG, middle	−48, −31, 4	4.77		
STG, posterior	−42, −43, 7	4.66		
MTG, middle	−57, −55, 16	3.82	2	
MTG, middle	−69, −40, 1	3.80	2	
STG, middle	−69, −10, 10	3.79	2	
**Right hemisphere**				
**STG, middle**	69, −7, −11	4.48	52	0.24
MTG, middle	66, −22, −11	4.42		
MTG, middle	69, −34, 1	3.70	1	

Peak voxel coordinates in standard MNI space and corresponding t-values above 3.85 (univariate analysis, FWE-corrected p < 0.05) and 3.70 for MVPA (FWE-corrected p < 0.05, as determined by permutation distribution with 10,000 permutations) and Cohen's d for large cluster size. The Cohen's d of the MVPA refers to the mean difference in classification accuracy (contrast estimates of the univariate analysis respectively), divided by the pooled standard deviation for those means.

The female > male contrast of classification accuracy maps identified significant voxel clusters in the middle part of the middle temporal gyrus (MTG) in both hemispheres, in which classification accuracy was greater for female than male participants (red clusters in Figure 2B). Areas of greater classification accuracy in females were more extended in the left hemisphere with an additional smaller cluster located in the STG. The peak voxels of female > male classification accuracy difference were located in the middle part of the MTG (bilateral), and the left middle STG (MNI coordinates left: *x* = −69, *y* = − 19, *z* = −8, cluster size 84, *T*-value = 5.22; *x* = −51, *y* = −22, *z* = 13, cluster size 156, *T*-value = 5.19; right: *x* = 69, *y* = −7, *z* = −11, cluster size 52, *T*-value = 4.48; cf. circle in Figure 2A). The Cohen's d effect size values (*d* = 0.35, 0.35, and 0.24) suggested a small difference at the peak voxel (Table 3). Classification accuracy (computed in native space) at these coordinates was distinctly above chance (50%) for both females and males, but higher in females across peaks (Figure 2B).

## Discussion

The present study aimed to investigate gender differences on voice localizer scans by employing the conventional univariate analysis as well as MVPA. Both analysis approaches revealed largely overlapping/comparable and robust estimates of the TVAs in female and male listeners. However, the MVPA was more sensitive to differences in the middle MTG of the left and right hemispheres and the middle left STG between genders as compared to univariate analysis with higher classification accuracy in women.

## Robust TVAs

The estimated TVAs using MVPA robustly replicated and confirmed prior fMRI findings applying the voice localizer (Belin et al., 2000, 2002; Belin and Zatorre, 2003; Scott and Johnsrude, 2003; Von Kriegstein et al., 2003). Both analysis methods showed comparable maps of classification accuracy (MVPA) and of vocal vs. non-vocal activity difference (univariate analysis) for both female and male listeners. The average classification accuracy at the peak voxel was distinctly above chance level and higher in female as compared to male listeners. The peak voxels were at comparable locations (along middle and posterior parts of the STS) for both analysis approaches and both genders. A small difference between the MVPA and univariate analysis can be seen bilateral at the temporal pole, where the MVPA detected more vocal/non-vocal differences in superficial cortical regions as compared to the univariate analysis. In addition to the activation brain maps showing the robustly estimated TVAs (univariate analysis), the MVPA results extend previous findings by providing a corresponding classification accuracy brain map. When brain maps are considered for each analysis approach and for female and male listeners separately, our findings showed no distinct differences between genders and between univariate analysis and MVPA. Instead comparable voxel clusters of a similar size in the bilateral temporal lobes were identified, verifying the prior univariate analysis and the robustness of the TVAs (see e.g., Belin et al., 2000).

## Gender differences

When data were analyzed with MVPA, differences between female and male listeners in response to vocal/non-vocal sounds were found by contrasting female > male (but not male > female). A significant difference in success of the MVPA between female and male listeners was apparent in the middle part of the MTG in both hemispheres and in the middle part of the STG in the left hemisphere. Effect sizes showed a small difference at the peak voxels. Despite the large sample size used in this study, the univariate analysis showed no major activation differences between genders. Only two small clusters with one to four voxels were significant in the posterior and anterior part of the STG. In the univariate analysis, the overall activation difference between vocal vs. non-vocal sounds was stronger in female as compared to male listeners and effect sizes showed a moderate difference at the peak voxels.

The distinct gender differences located in the middle part of MTG and middle part of STG between genders revealed by the MVPA survived our applied criteria (FWE-correction). In these regions, the classifier successfully distinguished between the vocal and non-vocal condition with better overall accuracy in females as compared to males across the peak voxels. Thus, BOLD signal in parts of auditory cortex seem to carry less information for discriminating vocal from nonvocal sounds in male than females listeners. We do not make any inference on the nature of the underlying processing differences in terms of mental states or cognitive mechanisms, but possible explanations for our findings are discussed below.

MVPA may overall be more sensitive to detect small differences in the activation patterns to vocal and non-vocal sounds. Thus, differences between genders appear significant only when analyzed with MVPA (Haynes et al., 2007; Kriegeskorte et al., 2006; Norman et al., 2006). The differences in classification accuracy between female and male listeners, identified in parts of auditory cortex, may be contributed to by a different predisposition of female/male listeners to the presented vocal sound samples of the voice localizer. Previous findings suggest a sex-difference in response to infant crying and laughing. Women showed a deactivation in the anterior cingulate cortex (ACC) to both laughing and crying (independent of parental status) as compared to men (Seifritz et al., 2003). In contrast, another study showed increased activation to infant vocalization in the amygdala and ACC whereas men showed increased activation to the control stimuli (fragment recombined and edge smoothed stimuli of the original laughing/crying samples). This may reflect a tendency in women for a response preference to infant vocal expressions (Sander et al., 2007). A recent study by De Pisapia et al. (2013) found a sex-difference in response to a baby cry. Women decreased brain activity in DPFC regions and posterior cingulate cortex when they suddenly and passively heard infant cries, whereas men did not. They interpreted their findings in such a way that the female brain interrupts on-going mind-wandering during cries and the male brain continues in self-reflection (De Pisapia et al., 2013). In our study half of the vocal stimuli consisted of infant vocalizations (also emotional expressions such as laughing and crying) and our results may reflect differences in the fine-grained pattern of distributed activity in female and male listeners in response to these vocal expressions of children and babies. The outcome in this study may be affected by anatomical differences in brain structure/size between female and male listeners (Brett et al., 2002). In general individuals vary in their anatomical brain structures and undergo the experiment with different mental states which may influence their brain responses (Huettel et al., 2008).

To date, there is also evidence for differences in the vocal processing and in particular in speech perception between genders from both behavioral (Hall, 1978; Skuk and Schweinberger, 2013) and previous fMRI studies (Shaywitz et al., 1995; Schirmer et al., 2002, 2004, 2007; Junger et al., 2013). These studies found activation differences in frontal brain regions (Schirmer et al., 2004; Junger et al., 2013) and the left posterior MTG and the angular gyrus (Junger et al., 2013). The deviation of the current results in terms of identified brain regions may be due to the different experimental design and computed contrasts, the different applied criteria (e.g., mask), number of included participants and implemented analysis methods. Future studies should further aim to elucidate the relationships between behavioral and functional activation differences. However, the current study shows that the choice of fMRI analysis method (e.g., MVPA) is of relevance when considering subtle between-gender differences.

Regarding the current study, it would be interesting to separate the different vocal categories in the analysis (e.g., by speaker: female/male adults vs. infants/babies) and to perform a behavioral task in order to link differences in brain activation to behavior of the listener. Furthermore, it would be interesting for future studies to take into account more specific aspects of voice quality, which were not considered in the current study. Even subtle differences in phonation (e.g., whispery voice, harshness of a voice), articulation (e.g., vowel space) and or prosody (e.g., pitch variability, loudness, tempo) are critical aspects of voice processing and could be investigated using similar methodical approaches. Apart from studying differences between women and men, also other listener characteristics, such as differences between young and elderly participants, different nationalities and/or familiarity with the presented voices/stimuli should be considered.

## Conclusion

Male and female participants were similar in their pattern of activity differences in response to vocal vs. nonvocal sounds in the TVA of the auditory cortex. Yet, MVPA revealed several regions of significant gender differences in classification performance between female and male listeners: in these regions the distributed pattern of local activity from female participants allowed significantly better vocal/nonvocal classification than that of male participants; no region showed the opposite male > female difference. The neuronal mechanims underlying the observed differences remain unclear.

### Conflict of interest statement

The authors declare that the research was conducted in the absence of any commercial or financial relationships that could be construed as a potential conflict of interest.
